# Evolution of Antiretroviral Drug Costs in Brazil in the Context of Free and Universal Access to AIDS Treatment 

**DOI:** 10.1371/journal.pmed.0040305

**Published:** 2007-11-13

**Authors:** Amy S Nunn, Elize M Fonseca, Francisco I Bastos, Sofia Gruskin, Joshua A Salomon

**Affiliations:** 1 Department of Population and International Health, Harvard School of Public Health, Boston, Massachusetts, United States of America; 2 Department of Health Information, Oswaldo Cruz Foundation, Rio de Janeiro, Brazil; 3 Department of Health Management and Policy, University of Michigan, Ann Arbor, Michigan, United States of America; 4 Program on International Health and Human Rights, Harvard School of Public Health, Boston, Massachusetts, United States of America; 5 Harvard University Initiative for Global Health, Cambridge, Massachusetts, United States of America; Hospital Sao Francisco de Assis, Brazil

## Abstract

**Background:**

Little is known about the long-term drug costs associated with treating AIDS in developing countries. Brazil's AIDS treatment program has been cited widely as the developing world's largest and most successful AIDS treatment program. The program guarantees free access to highly active antiretroviral therapy (HAART) for all people living with HIV/AIDS in need of treatment. Brazil produces non-patented generic antiretroviral drugs (ARVs), procures many patented ARVs with negotiated price reductions, and recently issued a compulsory license to import one patented ARV. In this study, we investigate the drivers of recent ARV cost trends in Brazil through analysis of drug-specific prices and expenditures between 2001 and 2005.

**Methods and Findings:**

We compared Brazil's ARV prices to those in other low- and middle-income countries. We analyzed trends in drug expenditures for HAART in Brazil from 2001 to 2005 on the basis of cost data disaggregated by each ARV purchased by the Brazilian program. We decomposed the overall changes in expenditures to compare the relative impacts of changes in drug prices and drug purchase quantities. We also estimated the excess costs attributable to the difference between prices for generics in Brazil and the lowest global prices for these drugs. Finally, we estimated the savings attributable to Brazil's reduced prices for patented drugs. Negotiated drug prices in Brazil are lowest for patented ARVs for which generic competition is emerging. In recent years, the prices for efavirenz and lopinavir–ritonavir (lopinavir/r) have been lower in Brazil than in other middle-income countries. In contrast, the price of tenofovir is US$200 higher per patient per year than that reported in other middle-income countries. Despite precipitous price declines for four patented ARVs, total Brazilian drug expenditures doubled, to reach US$414 million in 2005. We find that the major driver of cost increases was increased purchase quantities of six specific drugs: patented lopinavir/r, efavirenz, tenofovir, atazanavir, enfuvirtide, and a locally produced generic, fixed-dose combination of zidovudine and lamivudine (AZT/3TC). Because prices declined for many of the patented drugs that constitute the largest share of drug costs, nearly the entire increase in overall drug expenditures between 2001 and 2005 is attributable to increases in drug quantities. Had all drug quantities been held constant from 2001 until 2005 (or for those drugs entering treatment guidelines after 2001, held constant between the year of introduction and 2005), total costs would have increased by only an estimated US$7 million. We estimate that in the absence of price declines for patented drugs, Brazil would have spent a cumulative total of US$2 billion on drugs for HAART between 2001 and 2005, implying a savings of US$1.2 billion from price declines. Finally, in comparing Brazilian prices for locally produced generic ARVs to the lowest international prices meeting global pharmaceutical quality standards, we find that current prices for Brazil's locally produced generics are generally much higher than corresponding global prices, and note that these prices have risen in Brazil while declining globally. We estimate the excess costs of Brazil's locally produced generics totaled US$110 million from 2001 to 2005.

**Conclusions:**

Despite Brazil's more costly generic ARVs, the net result of ARV price changes has been a cost savings of approximately US$1 billion since 2001. HAART costs have nevertheless risen steeply as Brazil has scaled up treatment. These trends may foreshadow future AIDS treatment cost trends in other developing countries as more people start treatment, AIDS patients live longer and move from first-line to second and third-line treatment, AIDS treatment becomes more complex, generic competition emerges, and newer patented drugs become available. The specific application of the Brazilian model to other countries will depend, however, on the strength of their health systems, intellectual property regulations, epidemiological profiles, AIDS treatment guidelines, and differing capacities to produce drugs locally.

## Abstract 2

Amy Nunn and colleagues analyze the cost of antiretroviral drugs in Brazil between 2001 and 2005 and discuss the implications for HIV treatment in other developing countries.

## Editors' Summary

**Background.:**

Acquired immunodeficiency syndrome (AIDS) has killed 29 million people since the first case occurred in 1981 and an estimated 40 million people live with HIV/AIDS today. AIDS is caused by the human immunodeficiency virus (HIV), which destroys the immune system. Infected individuals are consequently very susceptible to other infections. Early in the AIDS epidemic, most HIV-positive individuals died within a few years of becoming infected. Then, in 1996, highly active antiretroviral therapy (HAART)—a cocktail of antiretroviral drugs (ARVs)—was developed. For people who could afford HAART (which holds HIV infections in check), AIDS became a chronic disease. People who start HAART must keep taking it or their illness will progress.

Unfortunately, few people in low- and middle-income countries could afford these expensive drugs. In 2001, ARV prices fell in developing countries as AIDS activists and developing country governments challenged pharmaceutical companies about ARV prices, pharmaceutical companies set tiered prices for the low- and middle-income countries and more generic (inexpensive copies of brand-named drugs) ARVs became available. In 2003, the lack of access to HIV/AIDS treatment was declared a global health emergency. Governments, international organizations, and funding bodies began to set targets and provide funds to increase access to HAART in developing countries. By 2007, over 2 million people in low- and middle-income countries had access to HAART, but another 5 million remain in urgent need of drugs for treatment.

**Why Was This Study Done?:**

In 1995, many countries in the world signed the World Trade Organization (WTO) Trade-Related Aspects of Intellectual Property (TRIPS) agreement, which requires countries to acknowledge intellectual property rights for many products, including pharmaceuticals. In 1996, Brazil became the first developing country to commit to and implement policies to provide free and universal access to HAART. Since then, Brazil's successful AIDS treatment program has become a model for the developing world, and 180,000 Brazilians were receiving HAART at the end of 2006. However, as a WTO member that signed on to the TRIPS agreement, Brazil was required to recognize the intellectual property rights of pharmaceutical companies' patented ARVs. As Brazil scaled up treatment in the late 1990s, the cost of treating AIDS patients rose quickly and the country took controversial public policy steps to reduce the cost of providing HAART to people living with HIV/AIDS. Brazil produces several non-patented ARVs locally, and since 2001 has challenged multinational pharmaceutical companies about the prices of patented ARVs. To induce price reductions for patented ARVs, Brazil has threatened to issue compulsory licenses (which under WTO terms allow countries facing a health emergency to produce patented drugs without consent of the company holding the patent). Brazil also recently issued a compulsory license for one ARV.

Although world leaders have set a target of universal access to HAART by 2010, little is known about the long-term costs of AIDS treatment in developing countries. In this study, the researchers have investigated how and why the costs of ARVs changed in Brazil between 2001 and 2005 and discuss the relevance of the Brazilian model for AIDS treatment for other resource-limited settings.

**What Did the Researchers Do and Find?:**

The researchers analyzed the prices for each ARV recommended in Brazil's therapeutic guidelines for adults and estimated the changes in purchase quantities for each between 2001 and 2005. These changes likely stem from the growing number of options in Brazil's treatment guidelines, the steadily rising number of patients commencing treatment, and patients' shifts to second- and third-line treatments when their HIV infection became resistant to first-line drugs or they developed side effects. The researchers report that the generic drugs produced in Brazil were generally more expensive than similar drugs made elsewhere, but Brazil's negotiated drug prices for many patented ARVs were lower than elsewhere. Overall, total annual drug expenditure on ARVs doubled between 2001 and 2005, reaching US$414 million in 2005. Because many drug prices fell sharply as a result of declining patented drug prices over the study period, this increase was mainly attributable to increases in drug quantities purchased. If these quantities had stayed constant, the total annual cost would have increased by only $7 million, to $211 million. Conversely, without the decrease in the price of patented drugs, Brazil would have spent $952 million annually by 2005. If Brazil had enjoyed the lowest global prices for generic medicines, the total costs per year in 2005 would have been $367 million, or nearly $50 million less than the costs Brazil actually realized.

**What Do These Findings Mean?:**

These findings tease out the many factors—clinical, commercial, and political—that affected the total costs of the Brazilian AIDS treatment program between 2001 and 2005.

Brazil's ability to produce generic drugs facilitated Brazil's price negotiations for patented drugs. Although Brazil saved approximately US$1 billion over the study period as a result of declining prices for patented medicines, the cost of producing generic drugs locally has risen while the prices for generic drugs have fallen elsewhere. Brazil's recent decision to import a generic ARV using a compulsory license suggests that the Brazilian model for AIDS treatment continues to evolve.

Questions remain about the precise causes of year-to-year cost trends in Brazil because, for example, the researchers did not have full data on when patients switched from first-line to second- or third-line drugs. The observed steep rise in costs from 2004 to 2005 in particular warrants further analysis. In addition, the findings may not be generalizable to countries with different policies on HIV/AIDS treatment, different access to generic drugs, or different bargaining power with multinational drug companies. Nevertheless, the trends this study highlights provide important information about how AIDS treatment costs are likely to evolve in other developing countries as efforts are made to provide universal access to life-saving ARVs.

**Additional Information.:**

Please access these Web sites via the online version of this summary at http://dx.doi.org/10.1371/journal.pmed.0040305.

- Information from the US National Institute of Allergy and Infectious Diseases on HIV infection and AIDS
- Information from the US Centers for Disease Control and Prevention on global HIV/AIDS topics (in English and Spanish)
- HIV InSite, comprehensive and up-to-date information on all aspects of HIV/AIDS from the University of California San Francisco
- Information from Avert, an international AIDS charity, on HIV and AIDS in Brazil and on HIV/AIDS treatment and care, including universal access to ARVs
- Progress towards universal access to HIV/AIDS treatment, the latest report from the World Health Organization (available in several languages)
- The National STD and AIDS Program of Brazil

## Introduction

The World Health Organization (WHO) estimates that globally, 2 million AIDS patients in developing countries were receiving highly active antiretroviral therapy (HAART) in December of 2006, a more than five-fold increase since 2001. However, this number is only about 26% of the estimated 7.1 million people needing HAART [1]. Because large-scale treatment began only recently in many developing countries, little is known about the long-term costs of drugs for AIDS treatment. Prices of many global first-line antiretroviral drugs (ARVs) recently declined precipitously with multinational pharmaceutical companies' tiered prices for low-, middle-, and high-income countries and new generic competition for ARVs [2]. However, even the lowest price—US$142 per person per year (PPPY) for the first-line HAART regimen—reported to the WHO Global Price Reporting Mechanism remains out of reach for many patients in resource-limited settings. As patients receive HAART for longer periods, AIDS case management becomes more complex and patients often require more expensive second- and third-line ARVs. Also, over time new ARVs have emerged, offering therapeutic improvements with fewer pills. Although the prices of some second-line ARVs have also declined in some countries, second-line treatment is nearly always more expensive than first-line treatment because of the high costs associated with developing new technologies and the monopoly prices innovator companies enjoy during patent terms [2]. As treatment scales up globally, many AIDS patients now receiving first-line therapies will need therapeutic alternatives. The cost of second- and third-line AIDS treatment and access to the latest ARV therapies is therefore a problem of global public health concern.

**Figure 1:**
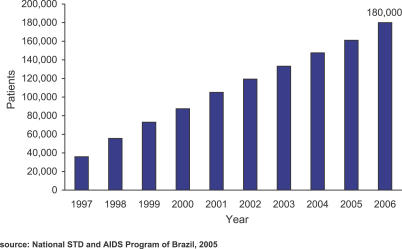
Patients Receiving HAART in Brazil, 1997–2005

Brazil, a middle-income country, has provided free and universal access to HAART since 1996 but is grappling with the rising cost of AIDS treatment. In Brazil, HAART is recommended to symptomatic HIV-positive patients regardless of viral load counts and to asymptomatic patients with CD4^+^ T cell counts below 200/mm^3^. HAART is considered for asymptomatic patients with CD4^+^ cell counts between 200/mm^3^ and 350/mm^3^; decisions to start treatment are determined on a case-by-case basis. The total number of patients receiving HAART has increased each year since 1997, with a reported 180,000 of the estimated 600,000 HIV-infected Brazilians receiving treatment in 2006 (Figure 1) (AIDS Care and Treatment Department of the National STD and AIDS Program of Brazil, personal communication) [3]. Over this period, mother-to-child transmission of HIV declined; AIDS-related hospitalizations, mortality, and morbidity declined; and life expectancy of AIDS patients more than tripled from an estimated 18 to 58 months [4–10]. Estimated AIDS incidence has plateaued, particularly in southeastern Brazil, the region with highest HIV prevalence [9,11]. Nonetheless, the number of patients receiving HAART is likely to increase as Brazil continues to expand treatment and patients live longer.

The cost of AIDS treatment in Brazil has grown in recent years as clinical, social, and political circumstances have evolved. In the 1990s, Brazil's Health Ministry began producing generic ARVs locally. However, in 1997, Brazil, which has the world's tenth largest pharmaceutical industry and a long history of public drug production, began to recognize intellectual property protection for pharmaceutical products. Patent legislation was adopted in Brazil much earlier than the World Trade Organization (WTO) deadlines of 2005 and 2016 for middle- and low-income countries [12–14]. As a result, 11 of the 18 drugs in Brazil's current treatment guidelines are now patented, and in several cases are more expensive in Brazil than in other developing countries. Brazil's Health Ministry still produces seven ARVs introduced before 1997. Since 2001, the Health Ministry has threatened to issue compulsory licenses in order to produce additional ARVs locally. Under WTO rules, a compulsory license allows governments to produce or to grant a third-party authority to produce a drug without consent of the patent holder in cases of national public health emergency, among other limited circumstances [12,14]. Brazil's threats to issue compulsory licenses have attracted international attention about ARV prices, prompted a trade dispute with the United States, and induced price negotiations with multinational pharmaceutical companies for five patented ARVs [15,16]. Together, Brazil's policies of providing universal access to treatment, producing generic ARVs, and negotiating prices for patented drugs have been referred to as the “Brazilian model” for AIDS treatment [17]. In May 2007, Brazil issued its first compulsory license for an ARV. Under its terms, Brazil imports generic efavirenz from drug companies other than the patent holder Merck and Company (hereafter Merck) [18]. The recent issuance of the compulsory license for efavirenz suggests that Brazil's AIDS treatment model continues to evolve.

Brazil's treatment guidelines generally include more ARVs than those issued by other developing countries. The variety of therapeutic options in use in Brazil reflects a combination of clinical and social factors. Clinical factors include the emergence and transmission of resistant HIV strains [19–21]; adverse events and side effects stemming from long-term AIDS treatment [22,23]; complex case management of AIDS coinfections such as hepatitis C and tuberculosis; and complications related to drug dependence and psychiatric disorders [24–26]. Social factors include pressure from civil society groups and AIDS patients to provide the newest ARVs and judicial decisions that stipulate that a constitutional right to health care includes access to new ARVs [27]. For these reasons, and the clear therapeutic and practical benefits of the new ARVs available in the international marketplace, Brazil has integrated new ARVs such as lopinavir–ritonavir (lopinavir/r), atazanavir, tenofovir, and enfuvirtide into treatment guidelines; purchase quantities of each of these patented drugs have increased significantly over time.

Recent reports have described aggregate-level cost trends in Brazil, noting in particular a sharp increase in total costs per treated patient between 2004 and 2005 [16,28]. In this study, we aim to unpack these overall trends by examining expenditure information disaggregated by specific drugs, in combination with information on evolving drug prices over time. We expand on a recent study examining HAART cost trends in Brazil [29] in several ways. First, we examine the clinical factors contributing to Brazil's rising HAART costs. Second, we quantify the economic consequences of Brazil's policies of producing generic drugs locally and negotiating price reductions for patented drugs. Third, we investigate the relative contributions of changes in quantities and prices of specific drugs to overall cost trends. Finally, we quantify how HAART costs might have differed under a set of important alternative assumptions.

## Methods

### Overview

For the purposes of this paper, we use the term “cost” to refer to total aggregate expenditure on one or more ARVs and “price” to refer to the per-pill or annual per-patient purchase price of a drug.

We analyzed drug prices from various sources (see “Data,” below) for the years 2001 to 2005 for each of the drugs in Brazil's therapeutic guidelines for adults [30]. These represent the most recent years for which accurate, detailed cost and price data were available. Pediatric drugs were excluded from this analysis because they are used with small numbers of patients and represent a small fraction of total expenditures. Generic names for ARVs are used for ease of international comparison. Pharmaceutical companies often offer tiered prices for low-, middle-, and high-income countries, but in some cases offer only two price tiers for low-income and “all other” countries. Brazil falls into the middle-income category when three tiers are listed and the “all other” category when two tiers are listed.

We estimated changes in the purchase quantities for each specific drug during this period based on reports of annual, drug-specific costs, combined with prices in Brazil for corresponding years. Using a series of counterfactual analyses, we decomposed cost trends into the components attributable to price and quantity changes. With these models, we estimated both excess costs and cost savings related to Brazilian prices for generic and patented drugs.

### Data

Brazil's National STD and AIDS Program provided official data on drug costs and prices for each ARV from 2001 to 2006 (AIDS Care and Treatment Department of the National STD and AIDS Program of Brazil, personal communications). For international comparisons, historical price data were compiled from a range of sources, including Doctors Without Borders' (Médecins Sans Frontières, MSF) pricing guides for each year from 2001 to 2006 [2,31–35]; the Clinton Foundation's Procurement Consortium August 2006 ARV price list [36]; and WHO's August 2006 Global Price Reporting Mechanism Report [37], which reports on ARV prices procured for numerous countries through UNICEF, the International Dispensary Association, the Global Fund to Fight AIDS, Tuberculosis and Malaria, and other leading nonprofit organizations that procure ARVs. Global prices for Bristol Meyers Squibb's atazanavir and Roche's enfuvirtide were not available from these sources and were requested but not provided by innovator companies. The 2007 prices for Abbott Laboratories' (hereafter Abbott) lopinavir/r were obtained from Abbott's Web site [38].

The Brazilian AIDS Program provided drug prices in nominal US dollars. We first adjusted for inflation by converting all drug prices into 2005 US dollars using the gross domestic product deflator series from the United States Department of Commerce [39]. We then converted per-pill prices to per patient per year (PPPY) prices based on clinical dosing guidelines and rounded up to the nearest dollar.

Drug costs were provided in Brazilian reals. Cost data were converted to US dollars using average annual exchange rates according to the United States Federal Reserve Bank's historical records [40]. All cost data were then converted to 2005 US dollars using gross domestic product deflators.

Tariffs, freight, and insurance generally account for 3%–15% increases over drug list prices [35]. The data we collected on prices reflect these charges to varying degrees. Brazil's prices do not include any shipping, insurance, or government taxes. WHO prices include transactions for ARVs during 2005; do not include taxes, tariffs, or freight charges; are weighted for volume; and do not include outliers or donations [37]. Clinton Foundation prices include shipping and insurance charges. MSF's generic prices and prices for patented lopinavir/r, ritonavir, saquinavir, and tenofovir do not include transport, freight, and insurance charges. Other MSF prices reflect these charges [32].

### Analysis

#### Price comparisons.

To compare drug prices in Brazil to those in other middle and low-income countries, we examined Brazil's 2006 PPPY prices alongside prices reported by the 2006 WHO Price Reporting Mechanism, the Clinton Foundation and MSF. Prices for patented ARVs in Brazil reflect price negotiations that have taken place since 1998. Reported percentage price declines for efavirenz and nelfinavir are based on the price differences between the year the drug was launched in Brazil and 2006 prices. Reported percentage price declines for lopinavir/r, atazanavir, and tenofovir are based on the initial price offered by the pharmaceutical company prior to the drug's launch in Brazil and 2006 prices. To constrain price comparisons to include only those drugs meeting international quality and efficacy standards, whenever possible we restricted our analysis to drugs that had been approved by either the WHO Prequalification System or the US Food and Drug Administration. Both agencies require bioequivalence testing and good manufacturing, laboratory, and clinical practices [41,42]. To make price comparisons for the last year for which data were available in Brazil, we ended our comparative price analysis in 2006.

#### Trends in HAART costs.

We analyzed HAART cost trends from 2001 to 2005 using annual costs for each drug in Brazil's treatment guidelines. We computed yearly ratios of total ARV costs to the numbers of patients on treatment from 2001 to 2005. Despite volatility in intra-year exchange rates during the period 2001–2005, we used average exchange rates in each year for clarity of presentation. To quantify the impact of exchange rate volatility on our estimates, we report costs and ranges about these costs based on the highest and lowest exchange rates for each year.

#### Contributions of changes in drug prices and quantities to cost trends.

To disaggregate the effects of changes in drug prices and quantities on costs, we first present a graphical analysis of the drug-specific prices and purchase quantities in each year from 2001 to 2005. The number of daily doses for each drug purchased in each year is computed by dividing the drug-specific costs in a given year by the price per dose in that year. For ease of presentation, we grouped generics other than zidovudine–lamivudine (AZT/3TC) into one category, AZT/3TC into another category, and patented drugs whose prices were not negotiated into another. For the first and third groups, we present the volume-weighted average daily price.

Next we use a series of counterfactual analyses to examine the contributions of changes in drug prices and purchase quantities to trends in overall costs and to compute cost savings and excess costs relating to price changes for specific drugs. In our first counterfactual scenario, we partitioned changes in total costs into the contributions of quantity changes and price changes by estimating total costs assuming the quantities of all drugs remained constant at their 2001 levels (or at the same level as in the year of introduction for those drugs entering treatment guidelines after 2001), but drug-specific prices changed as observed.

In a second counterfactual analysis, we computed the cost savings associated with price reductions for patented drugs in Brazil. In this scenario, drug quantities change as reported between 2001 and 2005, but the prices for patented ARVs remain constant at their 2001 levels over this period.

Finally, we modeled a third counterfactual scenario to estimate the excess costs of higher prices for generics in Brazil as compared to the lowest prices on the international market. In this counterfactual, we estimated the trends in total costs that would be expected if quantities of all drugs changed between 2001 and 2005 as observed but the generic prices in Brazil matched the lowest observed values on the international market. In this analysis, we only considered prices for prequalified drugs as reported by MSF. For example, although generic didanosine was available more cheaply outside Brazil, we did not include it in our analysis because it had not been prequalified by the US Food and Drug Administration or WHO.

## Results

### Price Comparisons

Brazil's 2006 prices for generic ARVs (including AZT/3TC) were higher than prices reported to the Global Price Reporting Mechanism and MSF and higher than prices enjoyed by countries in the Clinton Foundation Procurement Consortium. Prices for some generics in Brazil were two to four times higher than reported prices in other developing countries (Table 1).

**Table 1:**
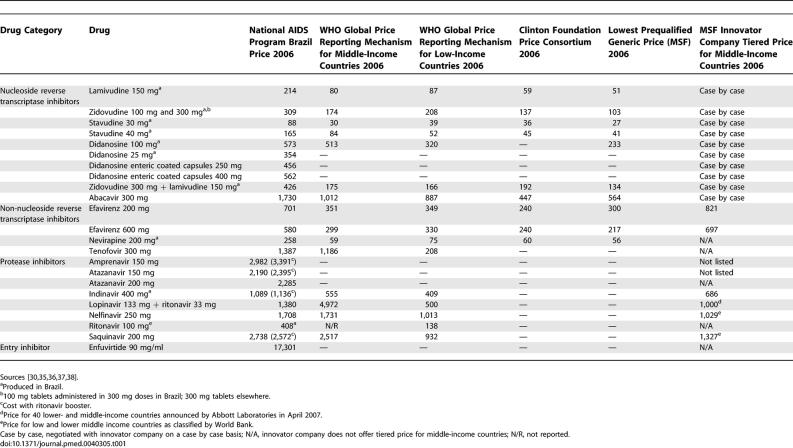
International Price Comparisons for ARVs in Brazil and Other Developing Countries, PPPY, US$

Prices PPPY for patented ARVs efavirenz and lopinavir/r were lower in Brazil than in other middle-income countries (Table 1). Brazil's 2006 price of US$1,380 for Abbott's lopinavir/r was 72% lower than prices for this drug in other middle-income countries reported to WHO. However, in April and July 2007, Abbott offered price reductions to 40 more low- and middle-income countries, which resulted in lower global prices for lopinavir/r in Brazil and elsewhere [38,43]. Brazil's US$580 PPPY efavirenz price was lower than Merck's US$697 price for middle-income countries with similar HIV prevalence rates, but higher than generic competitors and the average price of other middle-income countries. Even after the most recent price negotiation in Brazil with Gilead Sciences, the US$1,327 PPPY price of tenofovir was higher than the US$1,186 average middle-income country price reported to the WHO.

### Trends in HAART Costs

**Figure 2:**
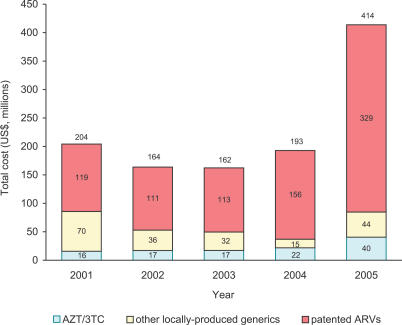
Total ARV Costs in Brazil, 2001–2005

**Figure 3:**
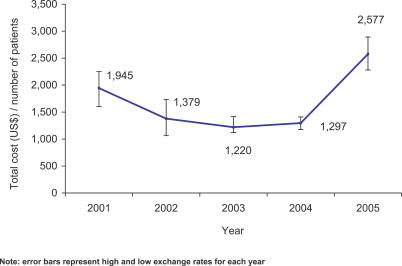
Ratio of Total Annual ARV Costs in US Dollars to Numbers of Patients on Treatment

Total HAART costs in Brazil declined from US$204 million in 2001 to US$162 million in 2003, before rising by around 20% in 2004 and then more than doubling in 2005 to US$414 million. Patented ARVs accounted for between 60% and 70% of total costs from 2001 to 2003 but then jumped to 80% of total costs in 2004 and 2005 (Figure 2). The substantial rise in total costs between 2004 and 2005 contrasts with the relatively steady yearly rise in patient numbers over this period (Figure 1). Consistent with previous reports [16,28] we found that the ratio of total costs to number of treated patients declined from US$1,945 in 2001 to US$1,220 in 2003 and then more than doubled to US$2,577 in 2005 (Figure 3). Assuming all drugs were consumed in the year of purchase, this ratio would be interpreted as the average treatment cost per person per year. In actuality, potential deviations from this perfect alignment of purchase and consumption times require that this ratio be interpreted cautiously. Though cost estimates varied slightly with intra-year exchange rates, trends were generally robust to exchange rate volatility.

Disaggregating costs by drug categories sheds some light on the drivers of broad cost trends. Generics declined as a share of total HAART cost from 2001 to 2005; they accounted for 42% of costs in 2001 (US$86 million) but only 20% (US$84 million) by 2005. Generic costs excluding AZT/3TC decreased from 34% of costs (US$70 million) in 2001 to 22% of costs (US$44 million) in 2005 (Figure 2). The declining relative contribution of generics is consistent with the substitution of more costly alternatives as therapeutic guidelines evolved over time. Indinavir, didanosine, and nevirapine were replaced by newer, more costly, patented first-line therapies such as lopinavir/r, atazanavir, tenofovir, and efavirenz. A fixed-dose combination of AZT/3TC replaced separate doses of the two drugs. Similarly, the patented protease inhibitors saquinavir and nelfinavir were replaced by more expensive but clinically preferable lopinavir/r and atazanavir in first- and second-line regimens [30,44]. However, we note that in absolute terms, costs associated with all categories of drugs actually increased between 2004 and 2005, and by a factor of two or more in some cases. These increases in all categories suggest that the sharp rise in total costs in 2005 is not explained simply by substitution of more expensive patented and generic drugs for less-expensive alternatives.

**Figure 4:**
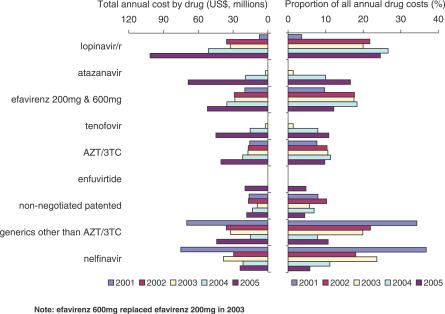
Total Annual Cost by Drug (Left) and Proportion of All Annual Drug Costs (Right)

Six ARVs with the greatest increases in costs over this period—enfuvirtide, AZT/3TC, tenofovir, efavirenz, atazanavir, and lopinavir/r—accounted for a US$284 million total increase from 2001 to 2005. Spending on these six drugs more than offset decreased spending on other ARVs during this period. Figure 4 displays the absolute and percentage growth of each of these six drugs' contributions to total costs.

### Contributions of Changes in Drug Price and Patient Volume to Cost Trends

The PPPY prices for several patented medicines, including efavirenz, nelfinavir, tenofovir, and lopinavir 133 mg with ritonavir 33 mg (one of two formulations of lopinavir/r used in Brazil during the period of analysis) declined over 50% from 2001 to 2005 (Table 2).

**Table 2:**
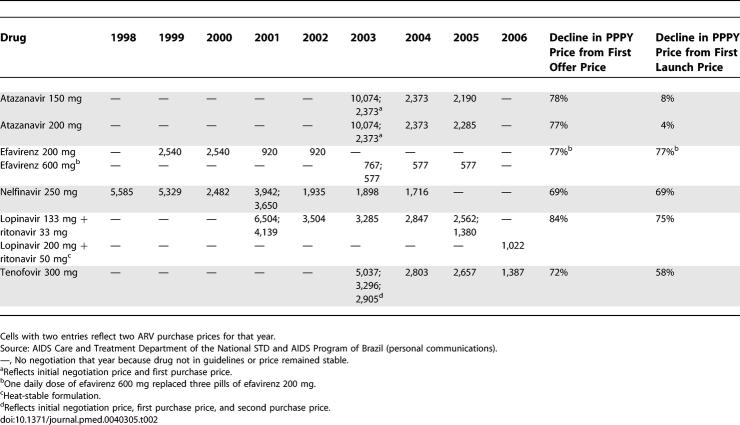
Price Declines for Select Patented ARVs in Brazil, PPPY, US$

**Figure 5:**
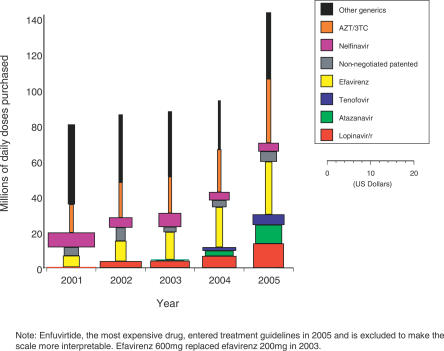
Contributions of Drug Price and Purchase Quantities to Total Cost, 2001–2005 The overall contribution of a particular drug to total costs in a given year is represented by the area of the rectangle that represents that drug. Changes in quantities for a particular drug appear as changes in the height of the rectangle. Changes in the price per daily dose of each ARV appear as changes in the width of the bars. The values associated with the width of the bars are reflected in the legend. The overall height of the bars in each year reflects the estimated total number of doses purchased in that year.

Trends in total costs may be decomposed into the fraction of the change attributable to changing drug prices and the fraction attributable to changing purchase quantities. Figure 5 offers a graphical illustration of the relative importance of prices and quantities to observed cost changes for each ARV from 2001 to 2005. Reinforcing the findings from analysis of total costs, we note that the sharp increase in the number of purchased doses in 2005 appears inconsistent with the rise that would be expected simply from patient scale-up, even allowing that some existing patients may have moved over time to regimens containing greater numbers of drugs.

For some drugs, such as AZT/3TC, changes in price were modest while quantities rose dramatically. For others, such as nelfinavir, both price and quantity declined over the period of analysis. For efavirenz and lopinavir/r, major price reductions were more than offset by substantial increases in volume from 2001 to 2005, and in particular from 2004 to 2005, thereby increasing the contributions of these drugs to overall costs. Tenofovir and atazanavir prices have declined moderately since they were added to treatment guidelines in 2003, but their volume rose rapidly from 2004 to 2005, contributing to substantial increases in total costs. Enfuvirtide prices have not been negotiated under the threat of issuing a compulsory license and its costs are relatively high despite limited quantities.

Price reductions and declining quantities of Roche's nelfinavir accounted for a US$37 million decline in total cost 2001–2003 (Figures 4 and 5). Total costs for locally produced generic indinavir and nevirapine also decreased by US$25 million from 2001 to 2003 as per-pill prices decreased 40% for indinavir and 58% for nevirapine (Figures 4 and 5). Other locally produced generics also accounted for an US$18 million decline in total costs because of falling per-pill generic prices. Together, these specific price and quantity reductions were the major drivers of observed declines in total costs from 2001 to 2003. Cost savings for patented nelfinavir and locally produced indinavir, nevirapine, and other generics offset cost increases for other drugs during the same time period.

In contrast to patented drugs whose prices have declined over time, per-pill prices for AZT/3TC and other locally produced generics have increased since 2003. The per-pill price of AZT/3TC decreased from US$0.50 in 2001 to US$0.42 in 2003 but then increased to US$0.56 in 2005. Similarly, the volume-weighted average price of other locally produced generics decreased from US$0.38 in 2001 to US$0.28 in 2003 but increased to US$0.37 in 2005 (Figure 5). Even after controlling for variation in exchange rates, per-pill generic drug prices still increased approximately 6%–7% each year between 2001 and 2004. From 2004 to 2005, less than 1% of the rising cost of per-pill generic prices was attributable to exchange rate variation.

### Counterfactual Analyses

**Figure 6:**
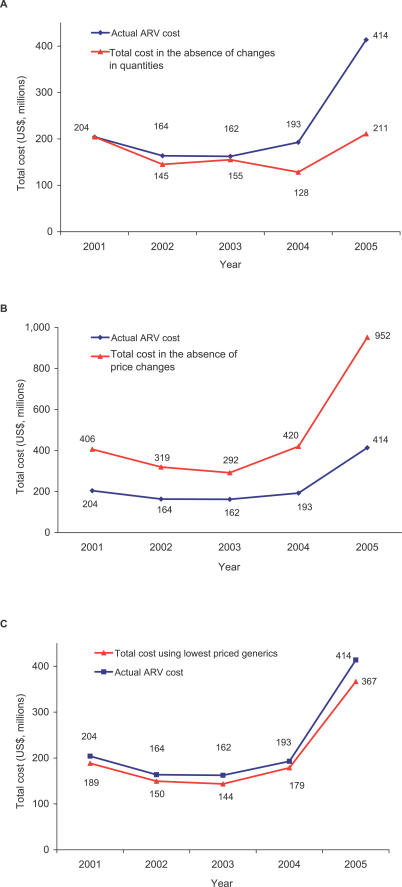
Impact of Alternative Price and Quantity Scenarios on Total ARV Costs, 2001–2005 (A) Total Cost Estimates in the Absence of Changes in Drug Quantities, 2001–2005. (B) Total Cost Estimates in the Absence of Changes in Drug Prices, 2001–2005. (C) Total Cost Estimates Using Lowest-Price Generics, 2001–2005.

The change in total costs between 2001 and 2005 is attributable almost entirely to changes in drug purchase quantities over this period. If quantities had remained constant between 2001 and 2005, total annual costs would have increased by only US$7 million between 2001 and 2005, and the cost in 2005 would have been US$203 million lower than the observed total, or approximately half as high (Figure 6A).

We estimate that the total cost savings resulting from price reductions for patented drugs was approximately US$1.2 billion from 2001 to 2005. Had patented drug prices remained constant over time, total drug costs would have been US$406 million rather than US$204 million in 2001 and would have reached US$952 million by 2005, more than twice as high as the observed 2005 total cost of US$414 million (Figure 6B). These estimated savings exceed Brazil's actual total ARV costs over this period.

We found, in contrast, that higher prices for generics in Brazil compared to the lowest prices on the international market produced a total excess cost of approximately US$110 million over this period, including an excess of US$47 million in 2005 alone. If Brazil had enjoyed the lowest prices for generics, total costs would have been US$189 million rather than US$204 million in 2001 and US$367 million rather than US$414 in 2005 (Figure 6C). In 2005, the bulk of savings would have stemmed from AZT/3TC, which accounted for only US$4 million (26%) of the hypothetical cost differential in 2001 but US$23 million (48%) in 2005.

## Discussion

Brazil's rapid progress in procuring and distributing the most modern ARVs to 180,000 AIDS patients is remarkable and has led to sustained improved health outcomes. We found that price reductions following negotiations with multinational pharmaceutical companies averted nearly half of the potential costs in the absence of these reductions, but that higher prices for generic drugs in Brazil forfeited another 10% in potential additional savings.

### Total Costs

Brazil's price negotiations with multinational pharmaceutical companies resulted in sustained price reductions for five patented ARVs from 2001 to 2005. Nonetheless, between 2003 and 2005, overall HAART costs increased by a factor of 2.5. While the total number of patients receiving HAART only increased by 14,000, or 8%, from 2004 to 2005, total costs more than doubled in this single-year interval alone. If we assumed that all ARVs purchased in a given year were consumed by patients treated during that year, the simple ratios of total costs to numbers of patients would imply a substantial jump in average per-patient drug expenditures [16,28,45]. However, our more detailed analyses caution against interpreting the 2005 rise in this way. By using data on drug-specific prices and costs to estimate purchase quantities of particular ARVs over time, we found that the attributions that have been postulated previously—for example, growing drug resistance [28]—are inadequate to fully explain the sudden and sharp spike in expenditures in 2005.

Decreases in total and per-patient ARV costs from 2001 to 2003 accompanied price negotiations for nelfinavir and declining per-pill prices for locally produced generics. However, as the standard of care has improved, nelfinavir and other locally produced generics have been replaced and supplemented with newer, patented ARVs in first-line treatment guidelines [30,45]. Even though Brazil's prices for the four patented drugs driving cost increases declined precipitously, this reduction was more than offset by increased purchase volumes of newer, patented ARVs and AZT/3TC, the main causes of cost increases in Brazil. Tenofovir and atazanavir were added to treatment guidelines in 2003 and scaled up to increasing numbers of patients as they were substituted for other drugs in 2004 and 2005. Similarly, in 2003 and 2004, lopinavir/r and efavirenz costs grew as they were increasingly substituted for other drugs in first-line and second-line regimens. Enfuvirtide was added as a new treatment option in 2005.

However, in disaggregating total costs by specific drugs, and parsing price changes from estimated changes in purchase volumes, we found that total costs and estimated purchase quantities actually increased for all categories of drugs between 2004 and 2005. Overall, we estimate that the total volume of drug doses purchased in 2005 was more than 50% higher than the volume purchased in 2004. These figures suggest that substitution of more expensive for less expensive alternatives is insufficient to explain the observed spike in total costs in 2005; moreover, the magnitude of the increase in total purchase volume remains difficult to reconcile with the increase in patient volume, even if some existing patients added more drugs to their treatment regimens as they developed resistance, experienced clinical failures or side effects associated with long-term treatment, or as formerly second-line therapies became first-line therapies. It is plausible that Brazil may have purchased more drugs in 2005 than in previous years in order to maintain adequate drug stocks during price negotiations. However, without more precise data or explanations about patient regimen changes—which we have requested but to date have not received from the Brazilian National STD and AIDS Program (AIDS Care and Treatment Department of the National STD and AIDS Program of Brazil, personal communications) definitive statements about the causes of the rise in costs in 2005 would be speculative.

### Cost Increases Reflect Therapeutic and Pragmatic Improvements

Though generally more expensive than previous first- and second-line drugs, the six ARVs responsible for total cost increases (enfuvirtide, AZT/3TC, tenofovir, efavirenz, atazanavir, and lopinavir/r) offer therapeutic benefits and pragmatic advantages over drugs included in Brazil's previous treatment guidelines. Newer ARVs often require fewer pills, may be effective in treatment-naïve and treatment-experienced patients, and tend to have fewer or different side effects [46–57]. For example, the 600 mg single-pill dosing of efavirenz is more convenient than the former 200 mg formulation and is US$120 cheaper PPPY. Similarly, the generic AZT/3TC fixed-dose combination is expected to enhance patient adherence to HAART by reducing pill volume [58–60]. At a price of US$17,301 PPPY, Roche/Trimeris' injectable entry inhibitor enfuvirtide is perhaps the most controversial drug in Brazil's treatment guidelines, but presents options for salvage therapy in highly treatment-experienced AIDS patients for whom other treatment options have failed [57].

### The Prices of Generics and Alternative Scenarios

In contrast with the international marketplace, where the prices of all generic ARVs have fallen substantially in recent years [31–35], Brazil's generic ARV prices have risen since 2003. With available information, it remains unclear why Brazil's prices are higher than generics that meet global drug quality standards produced in other countries.

A recent study attributed 75% of the cost of ARV production in Brazil and elsewhere to active pharmaceutical ingredients (APIs) and 25% to indirect costs [61]. Since Brazil's public drug production facilities only formulate drugs, do not conduct chemical synthesis to produce APIs, and import most APIs from China and India (Executive Office, Farmanguinhos Public Drug Production Facility, personal communications), the wide pricing differentials observed between generics manufactured in Brazil and elsewhere are surprising. The aforementioned article on HAART costs in Brazil attributes rising costs to “lack of investment in local production” but does not elaborate further [29]. Though requested, explanations about the causes of this trend were not clarified in interviews and other correspondence with several public officials who oversee drug policy and local drug production in Brazil (Executive Office, Farmanguinhos Public Drug Production Facility, Executive Office, Department of the National STD and AIDS Program of Brazil, personal communications). The former director of a public drug factory attributes the rising cost of generics to Brazil's public bidding process, which requires that the Health Ministry purchase the least expensive APIs, which do not always meet requisite quality standards. Low-quality or “impure” APIs must be replaced by higher-quality APIs, which ultimately raises total production costs. A recent newspaper article echoes this concern, but neither source clarifies why this trend emerged only recently (Farmanguinhos Public Drug Production Facility, personal communications) [62,71].

Irrespective of the cause of this trend in rising costs, lower international ARV prices suggest that other generic producers may have discovered important ways to lower either production or API costs, and that exploring the potential for cost savings for locally produced generics is both worthwhile and a potential avenue for new research. The potential inefficiency of local generic production is nevertheless dwarfed by Brazil's savings in price reductions for patented medicines. When Brazil's broader model is considered, accounting for both the relatively more costly locally produced generics and Brazil's reduced costs from price negotiations, Brazil still realized over US$1 billion in cost savings from 2001 to 2005.

Brazil's recent decision to issue a compulsory license and import generic efavirenz may signal an acknowledgement that other generic drug companies can now produce ARVs at much lower costs than Brazil's Health Ministry. However, Brazil might lose its ability to negotiate steep price reductions for patented ARVs if the Health Ministry stops producing generics. On the other hand, given the much lower prices elsewhere, Brazil may ultimately enjoy lower prices for patented ARVs by issuing compulsory licenses to import select drugs from generic pharmaceutical companies than by threatening to issue compulsory licenses to induce price reductions from innovator companies.

### Global Competition Lowers Brazil's Prices

Brazil generally has limited power to threaten to issue compulsory licenses and negotiate prices for drugs when no generics or APIs are available; often no generic competitors exist for several years after Brazil integrates the newest ARVs into treatment guidelines. Brazil's negotiations have therefore been most successful for ARVs for which generic competition is emerging, including lopinavir/r, efavirenz, and tenofovir, and less so for atazanavir, which does not yet have a WHO-prequalified generic competitor. Two recent examples highlight how generic competition has influenced global prices with direct effects on Brazil. First, in May 2006 Indian generic manufacturer Cipla launched a price of US$700 PPPY for generic tenofovir, which coincided with both Gilead's 50% price reduction for tenofovir in Brazil and Gilead's announcement that it would issue voluntary licenses to generic manufacturers to produce tenofovir [63]. (Under WTO trade rules, under voluntary license terms the patent holder grants third parties the right to produce drugs for a negotiated royalty fee [12,14].) Second, emerging competition also likely prompted Abbott's seven-year US$920 PPPY contract with Brazil for heat-stable lopinavir/r in 2005 and Abbott's 2007 decision to further lower lopinavir/r prices for 40 more low- and middle-income countries, including Brazil [38,64,65].

### Addressing Rising HAART Costs in Brazil

Brazil has achieved sustained reduced prices for several of the drugs for which generic APIs are available. Negotiating prices rather than issuing compulsory licenses has likely helped Brazil avoid further trade conflicts with the US and the WTO. The extent to which Brazil's recent decision to issue compulsory licenses will lower drug prices in Brazil may depend on whether Brazil produces the drugs or imports cheaper generics. If Brazil opts to produce its own generic versions of patented medicines and the cost of APIs declines with increased global demand and economies of scale, prices of select ARVs might decline further. However, given the observed rising costs of locally produced generics, it is unclear that issuing a compulsory license to produce generics locally would lower prices more than negotiating; Brazil's negotiated prices for patented drugs have historically been lower than prices for the same drugs in other middle-income countries, while Brazil's prices for locally produced generics have been higher. On the other hand, Brazil might enjoy significant cost savings if the Health Ministry imports the least-expensive generic ARVs available in the global marketplace. Whether Brazil imports or produces generic versions of patented medicines, Brazil's decision to issue compulsory licenses may have complex geopolitical consequences.

Despite rising costs, HAART expenditure as a percentage of total health expenditure represented a steady 2% of total annual health spending in Brazil from 2001 to 2005 [66]. Moreover, because overall public drug expenditure increased dramatically from 2001 to 2005 as a result of new public drug programs, HAART costs declined from 50% to 36% of total drug expenditure. These findings suggest that rising total HAART costs have not imperiled other public health spending. However, HAART costs dwarf drug spending for any one other disease, including those that account for a greater disease burden than HIV/AIDS in Brazil [66,67]. If HAART costs continue to increase rapidly, growth in HAART spending may eventually outpace growth in overall public health spending, which could detrimentally impact other public health programs.

Brazil is bound by law to provide drugs for all people living with HIV/AIDS (PLWHA) in need of treatment [27,68]. Brazil's courts have consistently interpreted this mandate liberally. As a result, and because of strong activist and Health Ministry support for Brazil's AIDS treatment policies, it is unlikely that Brazil will ration HAART or introduce means-testing for HIV/AIDS treatment, even if costs continue to increase. In light of these political realities and the findings we present, unless other companies dramatically lower their global ARV prices, Brazil's total HAART costs will likely rise as Brazil scales up treatment.

### Global Implications of Brazil's Model

Most developing countries enjoy much lower prices for the generic drugs Brazil produces locally; generic drug prices are therefore unlikely to contribute to rising costs to the same degree in other places as they have in Brazil. Though Brazil's initial prices for the newest ARVs may be higher than prices other developing countries ultimately enjoy for the same drugs, by threatening to issue or issuing compulsory licenses and producing drugs locally, Brazil has historically enjoyed lower prices for some ARVs than many other middle-income countries. While Brazil's model has been highly effective in lowering prices for patented ARVs, middle-income countries without domestic pharmaceutical industries or public drug production capacity have less power than Brazil to negotiate prices for patented drugs and may choose not to take the international political risks associated with issuing compulsory licenses. Moreover, even if other middle-income countries opt to issue compulsory licenses, importing generics may be cheaper and more feasible than producing drugs locally. Our cost findings may be less relevant to low-income countries, which typically enjoy the lowest global prices for patented ARVs but often do not integrate the most costly ARVs into treatment guidelines.

Brazil's model has affected ARV prices around the globe. First, Brazil's model set an important precedent for price negotiations and tiered pricing schemes for other developing countries. Second, Brazil's treatment policies have helped create a market for generic ARVs; in turn, generic competition has facilitated Brazil's price negotiations and lowered global ARV prices. Third, other countries have also used compulsory licenses in order to import drugs and reduce drug prices. For example, Thailand issued compulsory licenses for several antiretroviral and cardiovascular drugs in 2006 and 2007, including lopinavir/r and efavirenz, among others [65]. Thailand's decision to issue compulsory licenses, in turn, fostered greater transparency about global ARV prices and set a new precedent for middle-income countries. Shortly after Thailand issued compulsory licenses, Brazil issued its first compulsory license for Merck's efavirenz [18]. Additionally, in April 2007, Abbott further lowered its prices for original and heat-stable lopinavir/r from US$2,200 to US$1,000 PPPY in more than 40 lower middle-income and low-income countries (including Brazil and Thailand) and to US$500 for nine additional low-income countries outside sub-Saharan Africa [38,43].

### Study Limitations

We acknowledge several limitations to our study. First, because we did not have patient, pill, and cost data for 1998 to 2001, when prices for nelfinavir and efavirenz dropped considerably, we cannot estimate total cost savings from price declines prior to 2001. Similarly, some drug prices declined further after 2005, the last year for which cost data were available in Brazil. This study therefore could not assess the impact of 2006 and 2007 price declines on cost trends. Second, because prices are negotiated and ARVs are usually purchased up to six months prior to their consumption, there is not always a perfect match in reported annual costs and the number of patients taking each drug in a given year. The sharp spike noted above in total costs in 2005, which greatly exceeds the relative rise in patient numbers and might not be fully explained by individual regimen changes, may be due in part to this distortion in the timing of purchases relative to consumption. Further, drug prices from different sources vary slightly with costs of freight, shipping, taxes, and insurance. Because of data limitations and poor reporting on switches from first- to second- and third-line treatment, we were also unable to decompose our estimates of drug quantities attributable to patient volume versus drug mix. This article's findings therefore represent the most precise estimates currently available to our knowledge, rather than empirical absolutes.

### Conclusion

Brazil's price negotiations have been most effective in lowering costs for drugs in which generic competition has emerged. Despite declining patented ARV prices, Brazil's total HAART costs more than doubled since 2004. Cost increases reflect, in part, the progression of Brazil's AIDS epidemic ten years after introduction of free and universal access to HAART: more people began treatment, the standard of care evolved, and new drugs became available for both treatment-naïve and treatment-experienced patients. However, the incongruous rise in costs from 2004 to 2005 warrants further scrutiny. Brazil faces rising costs for many locally produced generic ARVs, particularly AZT/3TC. Brazil's AIDS treatment model nevertheless resulted in sustained lower prices for four of the six ARVs consuming the largest percentage of Brazil's HAART budget, saving Brazil over US$1 billion from 2001 to 2005.

The generalizability of these findings to other countries will depend on the strength of their health systems, treatment guidelines, intellectual property regimes, epidemiological profiles, approaches to scaling up AIDS treatment, differing capacities for local drug production, and on global drug prices, all of which continue to evolve. These findings nevertheless provide important lessons for other developing countries that aim to provide universal access to HAART. New therapeutically superior ARVs will become increasingly important components of global AIDS treatment guidelines as drug-resistant strains of HIV emerge in other low- and middle-income countries, patients develop side effects associated with long-term HAART, AIDS case management becomes more complex because of coinfections, and patients move from first- to second- and third-line treatment. As other middle- and low-income countries grapple with the challenges Brazil currently faces, they will have fewer options to directly negotiate ARV prices but may enjoy the fruits of generic ARV competition.

## Supporting Information

Alternative Language Abstract S1 Translation of the Abstract into Portuguese by Francisco Bastos, Amalia Bastos, and Amy Nunn (30 KB DOC)

Alternative Language Abstract S2 Translation of the Abstract into Spanish by Germán Velasco and Amy Nunn (41 KB DOC)

## Abbreviations

3TC: lamivudine
API: active pharmaceutical ingredient
ARV: antiretroviral drug
AZT: zidovudine
HAART: highly active antiretroviral treatment
PPPY: per person per year
WHO: World Health Organization
WTO: World Trade Organization
