# Supplementary material for: Evolution of Antiretroviral Drug Costs in Brazil in the Context of Free and Universal Access to AIDS Treatment
Source: PLoS Med. 2007 Nov 13;4(11):e305. doi: 10.1371/journal.pmed.0040305 (PMC2071936; doi:10.1371/journal.pmed.0040305)
Supplement: Supplementary file 1 [file pmed.0040305.sd001.doc]

**Evolução dos Custos dos Medicamentos Anti-retrovirais no Brasil no Contexto do Accesso Universal, Gratuito, ao Tratamento Anti-Aids**

**(Evolution of Antiretroviral Drug Costs in Brazil in the Context of Free and Universal Access to AIDS Treatment)**

**Resumo (Abstract)**

**Contexto (Background)**

Pouco se sabe sobre os custos de longo-prazo associados ao tratamento da AIDS nos países em desenvolvimento. O programa de acesso à terapia anti-AIDS do Brasil tem sido amplamente citado e é considerado o programa de acesso à terapia anti-AIDS mais amplo e bem sucedido entre todos os países em desenvolvimento no mundo. O programa garante acesso livre à terapia anti-retroviral de alta potência (HAART) para quaisquer pessoas vivendo com HIV/AIDS. O Brasil produz drogas anti-retrovirais genéricas, não-patenteadas, e adquire diversos anti-retrovirais (ARV) patenteados sob preços mais baixos, obtidos a partir de negociações, tendo, recentemente, procedido ao licenciamento compulsório de um ARV sob proteção patentária, que está sendo importado. No presente estudo, investigamos os fatores subjacentes às recentes tendências relativas aos custos dos ARV no Brasil, a partir de uma análise dos preços específicos de cada medicamento e dos respectivos gastos, entre os anos de 2001 e 2005.

**Métodos e Achados (Methods and Findings)**

Comparamos os preços dos ARV no Brasil aos de outros países de renda baixa e média. Analisamos as tendências dos gastos com medicamentos que integram a HAART, no Brasil, entre 2001 e 2005, a partir de custos desagregados por cada ARV adquirido pelo programa brasileiro. As mudanças gerais no âmbito dos gastos foram desagregadas, de modo a comparar os impactos relativos das mudanças referentes aos preços dos medicamentos e à quantidade de medicamentos adquiridos. Estimamos ainda o excedente de custos atribuídos à diferença entre os preços dos genéricos no Brasil frente aos preços globais mais baixos que é possível conseguir quanto a esses medicamentos. Finalmente, estimamos o quanto foi poupado em decorrência dos preços mais baixos dos medicamentos patenteados no Brasil.

Os medicamentos patenteados em uso no Brasil cujos preços são negociados se mostram mais baratos quando emerge a competição por meio dos genéricos. Em anos recentes, os preços do efavirenz e lopinavir/r têm sido mais baixos no Brasil do que em outros países de renda média. Por outro lado, o preço do tenofovir, por paciente, por ano, é, aproximadamente, R$ 400 (US$ 200) mais caro do que em outros países de renda média. A despeito do declínio pronunciado dos preços de quatro ARV patenteados, os gastos com medicamentos no Brasil dobraram, totalizando R$ 828 milhões (US$ 414 milhões), em 2005. Concluímos que os principais fatores que influenciaram os aumentos de custos foram as aquisições crescentes de seis medicamentos específicos: lopinavir/ritonavir (lopinavir/r), efavirenz, tenofovir, atazanavir, enfuvirtide (todos sob patente), além do medicamento produzido localmente em dose fixa, que resulta da combinação zidovudina / lamivudina (AZT/3TC).

Uma vez que os preços de muitos medicamentos patenteados que constituem a parcela maior dos gastos com a aquisição de medicamentos declinaram, praticamente todo o aumento de gastos agregados entre os anos de 2001 e 2005 pode ser atribuído a quantidades crescentes de medicamentos adquiridos. Caso todos os quantitativos referentes à aquisição de medicamentos tivessem se mantido constantes, de 2001 a 2005 (ou se para aqueles medicamentos incluídos nas diretrizes terapêuticas nacionais, adotadas após 2001, os quantitativos tivessem se mantido constantes entre o ano de sua introdução e 2005), os custos totais teriam aumentado em cerca de R$ 14 milhões (US$ 7 milhões), segundo nossas estimativas. Estimamos que, na ausência de um declínio de preço para drogas patenteadas, o Brasil teria gasto R$ 4 bilhões (US$ 2 bilhões) em medicamentos que integram a HAART, ao longo de 2001- 2005, o que se traduz em uma economia de R$ 2,4 bilhões (US$ 1.2 bilhões), decorrente do declínio dos preços dos medicamentos. Finalmente, comparando os preços brasileiros relativos aos genéricos produzidos localmente com os menores preços praticados internacionalmente que atendem aos padrões de qualidade farmacêutica global, concluímos que os preços atuais dos genéricos produzidos no Brasil são substancialmente mais elevados do que os preços globais correspondentes, cabendo observar ainda que estes preços têm aumentado no Brasil, enquanto diminuíam em todo o mundo. Estimamos que os custos adicionais dos genéricos produzidos no Brasil corresponderam a R$ 220 milhões (US$ 110 milhões), de 2001 a 2005.

**Conclusões (Conclusion)**

A despeito dos preços mais elevados dos ARV genéricos no Brasil, o balanço líquido dos gastos referentes a ARV aponta para uma economia de aproximadamente R$ 2 bilhões (US$ 1 bilhão), desde 2001. Os custos da HAART, no entanto, aumentaram acentuadamente, à medida que o Brasil ampliava o número de pessoas sob tratamento. Essas tendências podem ajudar a prever o futuro dos custos do tratamento da AIDS à medida que outros países em desenvolvimento vierem a incluir um número maior de pessoas em uso de ARV, os pacientes de AIDS viverem por um tempo mais longo e passarem de esquemas terapêuticos de primeira linha para esquemas terapêuticos de segunda e terceira linha, o tratamento de AIDS se tornar mais complexo, a competição com os genéricos emergir, e novas drogas patenteadas se tornarem disponíveis. A aplicação específica do modelo brasileiro aos demais países vai depender, no entanto, do vigor de seus sistemas de saúde, das normas referentes à propriedade intelectual, aos perfis epidemiológicos específicos, às diretrizes locais acerca do tratamento e da capacidade de cada país de produzir seus próprios medicamentos.
