# Supplementary material for: Evolution of Antiretroviral Drug Costs in Brazil in the Context of Free and Universal Access to AIDS Treatment
Source: PLoS Med. 2007 Nov 13;4(11):e305. doi: 10.1371/journal.pmed.0040305 (PMC2071936; doi:10.1371/journal.pmed.0040305)
Supplement: Supplementary file 2 [file pmed.0040305.sd002.doc]

**(Evolution of Antiretroviral Drug Costs in Brazil in the Context of Free and Universal Access to AIDS Treatment)**

Evolución de los Costos de Drogas Antiretrovirales en Brasil en el Contexto de Acceso Gratis y Universal para el Tratamiento del SIDA

**Introducción**

Poco se sabe acerca de los costos a largo plazo asociados con el tratamiento del SIDA en países en desarrollo. El programa brasilero del tratamiento del SIDA ha sido ampliamente citado como el programa más grande y exitoso del tratamiento del SIDA en los países en desarrollo. El programa garantiza acceso gratuito a terapia con antiretrovirales (ARV) a todas las personas que viven con el VIH/SIDA. Brasil produce drogas genéricas de ARV no-patentados, compra muchos ARV patentados por medio de negociaciones de reducción de precios y recientemente aprobó una licencia obligatoria para importar un ARV patentado. En este estudio, investigamos los factores responsables de las recientes tendencias de costos de ARV en Brasil por medio del análisis de precios específicos por droga y gastos entre el año 2001 y 2005.

**Métodos y Resultados**

Comparamos los precios de ARV brasileros con aquellos de otros países de ingreso medio y bajo. Analizamos las tendencias en los gastos de drogas para ARV en Brasil entre el año 2001 y 2005 basados en los datos de costos desagregados para cada uno de los ARV adquiridos por el programa brasilero. Descompusimos los cambios en gastos para comparar los impactos relativos de los cambios en precios de drogas y cantidades de drogas adquiridas. También estimamos el exceso de costos atribuidos a la diferencia entre los precios de drogas genéricas en Brasil comparados con los precios globales más bajos para estas drogas. Finalmente, estimamos los ahorros atribuidos a la reducción de precios en Brasil de drogas patentadas.

En Brasil, los precios negociados son más bajos para ARV patentados en los que competición genérica esta emergiendo. En los últimos años, los precios de efavirenz y lopinavir/r han sido más bajos en Brasil que en otros países de ingreso medio. Por otro lado, el precio de tenofovir es $200 dólares más alto por paciente por año que los precios reportados en otros países de ingreso medio. A pesar de la precipitada disminución de los precios de cuatro ARV patentados, el total de los gastos brasileros en drogas se ha duplicado, alcanzando $14 millones de dólares en el año 2005. Encontramos que el principal factor de aumento de los costos es el aumento de adquisición de seis drogas especificas: lopinavir/r, efavirenz, tenofovir, atazanavir, enfuvirtide (todas patentadas) y un genérico producido localmente, el cual es una combinación de zidovudine/lamivudine (AZT/3TC).

Debido a que los precios de muchas de las drogas patentadas disminuyeron, las cuales constituyen la mayor parte de los costos de drogas, casi que todo el aumento de los gastos en drogas entre el año 2001 y 2005 se atribuye al aumento en cantidades de drogas adquiridas. Si se hubieran mantenido constantes todas las cantidades de drogas adquiridas desde el año 2001 hasta el 2005 (o para aquellas drogas incluidas en las guías de tratamiento después del 2001, manteniendose constantes entre el año de introducción y el 2005) los costos totales hubieron aumentado solamente a un estimado de $7 millones de dólares. Estimamos que en la ausencia de una disminución de precios en las drogas patentadas, Brasil hubiera gastado un acumulado total de $2 billones de dólares en ARV entre el año 2001 y 2005, implicando ahorros de $1.2 billones de dólares debido a la disminución de precios. Finalmente, comparando los precios brasileros con los precios internactionales más bajos de drogas que han alcanzado estandares de calidad farmacéutica global, encontramos que los precios actuales de genériocs producidos localmente en Brasil son generalmente más altos que los precios globales. Se anota que estos precios han aumentado en Brasil mientras que han disminuido globalmente. Estimamos que el exceso de costo de los genéricos producidos localmente en Brasil totaliza $110 millones de dólares desde el año 2001 al 2005.

**Conclusiones**

A pesar de que los ARV genéricos brasileros son más costosos, el resultado neto de los cambios de precio ha sido un ahorro de aproximadamente $1 billón de dólares desde el año 2001. Sin embargo, los costos de tratamiento con ARV han estado escalando al mismo tiempo que Brasil ha aumentado la cobertura de tratamiento. Estas tendencias pueden ensombrecer los futuros costos de tratamiento con ARV en otros países en desarrollo a la vez en que se empiecen a tratar más personas, los pacientes con SIDA vivan más años, cambien de primera a segunda línea de tratamiento, los tratamientos del SIDA se vuelvan más complejos, la competición genérica emerja, y nuevas drogas patentadas se vuelvan disponibles. La aplicación específica del modelo brasilero a otros países dependerá, sin embargo, en la fuerza y el poder de los sistemas de salud, regulaciones de propiedad intelectual, tendencias epidemiológicas, guías de tratamiento del SIDA, y las diferentes capacidades de países para producir drogas localmente.
